# Prevalence of Comorbidities among Patients with Type-2 Diabetes Mellitus in Saudi Population

**DOI:** 10.12669/pjms.40.8.9003

**Published:** 2024-09

**Authors:** Kamran Sattar, Syed Irfan Karim, Ashfaq Akram, Fahad Abdulaziz Alrashed

**Affiliations:** 1 Kamran Sattar, MBBS, MMEd, PhD Department of Medical Education, College of Medicine, King Saud University, Riyadh, Saudi Arabia; 2 Syed Irfan Karim, MBBS, FRCGP Department of Family and Community Medicine, College of Medicine, King Saud University, Riyadh, Saudi Arabia; 3 Ashfaq Akram, BDS, MMEd, PhD Department of Medical Education, College of Medicine, King Saud University, Riyadh, Saudi Arabia; 4 Fahad Abdulaziz Alrashed, PhD Department of Medical Education, College of Medicine, King Saud University, Riyadh, Saudi Arabia

**Keywords:** Diabetes Mellitus, Cholesterol, Hypertension, Weight gain, HbA1c

## Abstract

**Objective:**

This study sought to quantify the link between low density lipoprotein (LDL) and cholesterol, glycemic control as measured by Hemoglobin A1c (HbA1c) level, and the impact of weight changes on subsequent risk of chronic heart disease in type-2 diabetes mellitus patients.

**Methods:**

This study was conducted at Primary Care Clinics from April to September 2023. The data were retrieved from the e-SiHi (a patient care management system). Appropriate parametric tests and non-parametric analysis were applied following the normality of the data.

**Results:**

In diabetes mellitus (DM) patients, the Pearson correlation between the cholesterol and LDL relationship was found to be strongly positive and statistically significant (r = .877, p <.001). HbA1c and cholesterol were moderately positive and statistically significant (r =.330, *p* =.003). Pearson correlation between DM and weight was found to be positive and statistically significant (r =.212, *p* =.05). Chi-square analysis showed an association of DM with Hypertension, and this association was also significant; X^2^ (1, n = 83; 11.877; *P* <.001).

**Conclusion:**

Diabetes mellitus regardless of gender, has a strong association with hypertension and weight gain. In DM patients, cholesterol and HbA1c are positively correlated. There is a strong need that primary care physicians should persistently advise for lifestyle changes in all their consultations with DM patients.

## INTRODUCTION

In all nations, regardless of their level of development, the prevalence of diabetes mellitus is rising. In the recent two decades, diabetes mellitus has doubled worldwide; therefore, it has become an essential challenge to public health.^1^ It has been discovered that diabetes mellitus is a major factor in the development of kidney diseases, heart attacks, strokes, blindness, and amputations of lower limbs additionally, age-specific diabetes mortality rates increased by 3% between 2000 and 2019 and estimated that diabetes-related kidney impairment was responsible for the deaths of approximately two million persons in 2019.^2^ Over the past 30 years, a lot of studies have been done on the relationship between Type-2 diabetes mellitus (T2DM) and cardiovascular disease (CVD), namely the Framingham study, which found that people with T2DM had a superior risk of CVD than those without T2DM.^3^

In Saudi Arabia, the frequency of diabetes is a major cause of morbidity and mortality. According to the World Health Organisation (WHO), Saudi Arabia has the second highest incidence of diabetes diagnoses in the Middle East and ranks seventh internationally in terms of the prevalence of diabetes.^4^ Three million people have prediabetes, and seven million individuals have diabetes.^5^ We calculated the overall frequency of diabetes in Saudi Arabia to be 23.7% built on a national study. At 27.9% and 26.4%, respectively, the northern and eastern regions had the highest incidence. The prevalence was 24.7, 23.7, and 18.2%, respectively, in the western, central, and southern regions.^6^ Compared to standard care, a randomized controlled parallel study of Scandinavian patients with co-morbid diabetes and early CKD (microalbuminuria) used multifaceted care (behaviour modification and drug therapy targeting hyperglycemia, hypertension, dyslipidemia, and microalbuminuria) and reported a decrease in albuminuria, retinopathy, neuropathy, and a composite outcome of CVD events or death.^7^,^8^

It has been a well-known understanding in primary care setup that a multidisciplinary approach in diabetes mellitus patients has been the cornerstone modality now. Any patients with Diabetes mellitus should be taken care by a multidisciplinary team rather than by a single specialist.^9^ Diabetic patients who seek primary care and present with co-morbidities such as hypertension and dyslipidemia typically experience minimal influence on their daily symptoms. In order to improve the delivery of health care for co-morbid conditions associated with diabetes, primary care physicians must be involved, and any obstacles in this path must identified and overcome.^10^ Obesity is a well-established predictor of Coronary Heart Disease (CHD) in the general population.^11^ Surprisingly, most studies of diabetics showed no link between obesity and CHD death or total mortality. These null relationships may be explained by the limited sample size and short follow-up period. Although weight growth is an independent risk factor for CHD, the link between the change in weight and the risk of CHD has not been well defined in the diabetic community.

HbA1c is a necessary marker to reflect the glycemic history of the previous three months. HbA1c also correlates well with the risk of long-term diabetes complications. Persistent high levels of HbA1c have also been regarded as an independent risk factor for coronary heart disease and stroke in subjects with diabetes mellitus. The information provided by a single HbA1c test has rendered it as a reliable biomarker for the diagnosis and prognosis of diabetes.^12^

Researchers have looked into the patterns of comorbidities that are present in Type-2 diabetes mellitus patients who visit a primary care clinic. It has been observed that the quality of care required to improve diabetes-related healthcare outcomes is complicated by the presence of comorbidities, which leads to financial loss for all stakeholders. The objective of this investigation was to assess the frequency of multimorbidity within the population of individuals with Type-2 diabetes who are under primary care. In addition, this study examined the impact of obesity and weight fluctuations on patients with Type-2 diabetes receiving primary care patients’ risk of developing chronic heart disease in the future. Glycemic control was measured by HbA1c level.

## METHODS

We carried out this study at Primary Care Clinics, at King Saud University Medical City (KSUMC) - from April to September, 2023. All protocols for regular follow-up appointments were followed to enter the department, examine the patients and give the treatments. The data pertaining to patients diagnosed with T2DM, who sought medical attention during the specified time frame were gathered. Every participant in this study had to go to the outpatient clinics at least once a year. At visits to the King Saud University Medical City (KSUMC), patients were asked to donate venous blood following an overnight fast of eight to ten hours. The serum was separated from the venous blood and stored at -80°C before inspection.

### Ethical Approval:

It was obtained from the IRB of the hospital. Ref. (IRB # E-23-8179; dated October 10, 2023).

The researcher IK had access to e-SiHi, an electronic application of patients’ data in the King Saud Medical City Hospital. The patients diagnosed with Type-2 and Type-1 DM were recognized in this study based on the ICD-10 codes E11, E11.901, and E11.902. The date of the first noted T2DM diagnosis was defined as the index date. Pre-index-date patient information was used to derive baseline data on demographic and illness characteristics. CVS diseases after the index date were labeled established on the ICD-10 codes E16.01, E16.001, E16.101, and E16.201, or a laboratory-measured glucose level of ≤ 3.9 mmol/L. Post-index-date diabetes-specific HCRU variables comprised the number of medical visits and hospitalizations. Post-index-date health care costs involved medical costs and prescription drug costs.

### Electronic health information system (e-SiHi):

The electronic medical records from the KSUMC database were processed using the da Vinci S^®^ system (provided by e-SiHi, Pearland, TX, USA). e-SiHi is an electronic health information system used and maintained by healthcare systems to collect and store patients’ medical information. This system is used across clinical care and healthcare administration to record a variety of medical information from individual patients over time, as well as to manage clinical workflows. This health information system contains different types of patient-level variables, such as demographics, diagnoses, problem lists, medications, vital signs, and laboratory data.^13^

The database held all of the clinical information for the hospital. The records included the date of admission, diagnosis, medication, testing, surgical data (if any) as well as images. In both the populations with CVS and neuropathy, we calculated the comorbidities related to diabetes problems.

### Statistical Characteristics:

The collected data consisted of mostly scale data, there were nominal data also. The normality of the data was weighed by means of Schapiro Wilk and Kolmogorov Smirnov. Scale data were presented with Mean and SD with minimum and maximum values. The nominal data were analyzed with Chi-square and the association of the various variables was presented with Chi-Square with *df* and *p-values*. Since many variables had been associated, therefore, Pearson correlation proceeded with *r* and *p-values*.

## RESULTS

The patients who had been identified with a diagnosis of diabetes either Type-1 or Type-2 were included. A total of 83 patients with diabetes mellitus (Type-I & Type-II) underwent the coding process through the e-SiHi platform. The percentage of T2DM (n=65, (78.3%) was much higher than Type-1 (n=18 (21.7%) (Table-Ia). The patients were diagnosed with DM ranging from one year to more than 20 years. A higher percentage of those patients (n = 15; 37.5 %) had been diagnosed with DM from 5-9 years (Table-Ib). From the demographic data, it was observed the mean weight of male patients was (Mean = 80.40, SD = 15.738) whereas the mean weight (KG) of female patients was (Mean = 79.88; SD= 20.184). The mean height of male and female patients was (Mean = 157.00; SD = 5.634; for males and Mean = 155.00, SD= 6.032 for females) respectively. The mean values of systolic and diastolic blood pressure are also shown in (Table-Ic).

**Table-Ia:** Percentage of diabetes mellites patients at the Department of Family Medicine in 2022.

Gender	n	Diabetes Type		Total

		Type-1	Type-2	
Male	Count	10	30	40
	Percentage within Gender	25.0%	75.0%	100.0%
Female	Count	8	35	43
	Percentage within Gender	18.6%	81.4%	100.0%
Total	Count	18	65	83
	Percentage within Gender	21.7%	78.3%	100.0%

**Table-Ib:** Distribution of diabetes mellitus patients in terms of duration.

Gender	n	Years (Diagnosis Made -DM)					Total

		1-4	5-9	10-15	16-20	21≥	
Male	Count	3	15	14	1	7	40
	Percentage within Gender Rev	7.5%	37.5%	35.0%	2.5%	17.5%	100.0%
Female	Count	8	5	12	7	11	43
	Percentage within Gender Rev	18.6%	11.6%	27.9%	16.3%	25.6%	100.0%
Total	Count	11	20	26	8	18	83
	Percentage within Gender Rev	13.3%	24.1%	31.3%	9.6%	21.7%	100.0%

**Table-Ic:** Mean values of weight, height, and blood pressure.

Gender	Variables	Mean	SD	Median	Minimum	Maximum
Male	Weight	80.40	15.738	80.50	52	118
	Height	157.23	5.634	168.00	152	182
	Systolic BP	132.78	17.183	130.50	100	177
	Diastolic BP	76.70	8.710	74.50	63	95
Female	Weight	79.88	20.184	77.00	55	138
	Height	155.00	6.032	156.00	144	171
	Systolic BP	133.49	14.549	135.00	98	164
	Diastolic BP	75.33	9.120	78.00	50	94

The values of Shapiro Wilk (*p*> .05) and Kolmogorov Smirnov (*p*>.05) for weight, height, and blood pressure show that data were normally distributed. However, the P value of shapiro-wilk for the weight of female patients was *p* <.001 showing this data were slightly skewed (Table-II).

**Table-II:** Normality of Data.

Variables	Gender	Kolmogorov-Smirnov^a^			Shapiro-Wilk		

		Statistic	Df	Sig.	Statistic	df	Sig.
Weight (Kg)	Male	.089	37	.200^*^	.965	37	.298
	Female	.167	43	.004	.891	43	.001
Height (Cm)	Male	.127	37	.139	.931	37	.024
	Female	.101	43	.200^*^	.971	43	.346
Blood Pressure Systolic	Male	.106	37	.200^*^	.967	37	.326
	Female	.119	43	.144	.970	43	.327
Blood Pressure Diastolic	Male	.125	37	.157	.960	37	.195
	Female	.127	43	.079	.976	43	.489

Pearson correlation between low-density lipoprotein (LDL) and cholesterol was found strongly positive and statistically significant (r = .877, *p* <.001. HbA1c and cholesterol were slightly moderately positive and statistically significant (r =.330, *p* =.003). Hence, H_1_ was supported. Similarly, this shows that an increase in cholesterol level would be more likely to have a high level of LDL. However, there was no significant correlation between the triglyceride of the person and urine albuminuria (*p* >.05) (Table-IIIa).

**Table-IIIa:** Correlation of multiple variables in diabetic patients.

		HBA1C	Cholesterol	LDL	HDL	Triglyceride	Urine Albuminuria
HBA1C	Pearson Correlation	1					
	Sig. (2-tailed)						
	N	82					
Cholesterol	Pearson Correlation	.330**	1				
	Sig. (2-tailed)	.003					
	N	81	82				
LDL	Pearson Correlation	.335**	.877**	1			
	Sig. (2-tailed)	.002	.000				
	N	81	82	82			
HDL	Pearson Correlation	-.041	.130	.002	1		
	Sig. (2-tailed)	.719	.243	.987			
	N	81	82	82	82		
Triglyceride	Pearson Correlation	.109	.145	-.033	-.294**	1	
	Sig. (2-tailed)	.334	.196	.773	.008		
	N	80	81	81	81	81	
Urine Albuminuria	Pearson Correlation	.041	.078	-.109	-.166	.044	1
	Sig. (2-tailed)	.723	.500	.342	.147	.705	
	N	77	78	78	78	77	78

** Correlation is significant at the 0.01 level (2-tailed).

Pearson correlation between DM and weight was found to be positive and statistically significant (r =.212, *p* =.05). Hence, H_1_ was supported. This shows that a heavy-weight person would be more likely to develop diabetes. However, there was no significant correlation between the height of the person and diabetes development (r =.106, *p* =.338) (Table-IIIb).

**Table-IIIb:** Correlation of weight, height, and diabetes mellitus.

Variables		Diabetes	Height	Weight
Diabetes	Pearson Correlation	1		
	Sig. (2-tailed)			
	N	83		
Height	Pearson Correlation	.106	1	
	Sig. (2-tailed)	.338		
	N	83	83	
Weight	Pearson Correlation	.212	-.051	1
	Sig. (2-tailed)	.055	.647	
	N	83	83	83

A chi-square test of independence was performed to observe the relationship between diabetes Mellitus and its association with hypertension. It was found there is an association of DM with hypertension and this association was significant; X^2^ (1, n = 83; 11.877; *p* <.001). There were more Diabetic patients likely to have hypertension (Table-IVa). A chi-square test of independence was performed to observe the relationship between diabetes mellitus and its association with cardiovascular diseases. It was found a weak association of DM with CVD and it was not significant; X^2^ (1, n = 72; .506; *p* =.477) (Table-IVb).

**Table-IVa:** Association of blood pressure with diabetes mellitus.

Blood Pressure	Diabetes Type		Total	df	X^2^	P value

	Type 1	Type 2				
Count (NO)	16	28	44	1	11.877	.001
Percentage within Hypertension	36.4%	63.6%	100.0%			
Count (YES)	2	37	39			
Percentage within Hypertension	5.1%	94.9%	100.0%			
Count (Total)	18	65	83			
Percentage within Hypertension	21.7%	78.3%	100.0%			

**Table-IVb:** Association of diabetes mellitus with cardiovascular diseases.

Diabetes Type & CVD	Type 1	Type 2	Total	df	X^2^	P value
Count (NO)	8	27	35	1	.506	.477
Percentage within Cardiovascular _DM	22.9%	77.1%	100.0%			
Count (YES)	6	31	37			
Percentage within Cardiovascular _DM	16.2%	83.8%	100.0%			
Count (Total)	14	58	72			
Percentage within Cardiovascular _DM	19.4%	80.6%	100.0%			

## DISCUSSION

In this study, we studied the patterns of comorbidities that patients experience with T2DM when visiting primary care clinics. Weight control in T2DM is the first step toward achieving glucose control. This study has several advantages. The majority of studies in the past were on longitudinal weight changes and used information-based questionnaires to determine self-reported body weight or previous weight loss events. The data in our study was derived from the physical examination performed during the study participants’ health screenings and we employed genuine measurements of weight variables.

According to studies, a very low-calorie liquid diet has been linked with radical weight loss during the first eight years of diabetes mellitus and recovery of normal glucose tolerance. Even gastric weight loss surgeries especially by the Roux-en-Y- method have played a significant role in overweight individuals.^14^ The mechanism behind this procedure is to remove the ectopic fat in and around pancreatic islets of Langerhans by decreasing its pro-inflammatory effect.^15^ For the proper management of T2DM patients, it is pivotal to understand the relation between weight change and the risk of CVD provided that obesity is relatively prevalent among them weight control is the key component.

In another study a significant association was found between T2DM and cardiovascular diseases (dyslipidemia, peripheral artery diseases, heart failure, stroke, coronary artery diseases, and Hypertension), causing mortality in 50% of these patients.^4^ It has also been found that keeping blood pressure between 120 and 70 mm Hg could further reduce macrovascular, and microvascular events and overall mortality.^16^ It is widely acknowledged that reducing blood pressure has a beneficial effect on CVD events and microvascular complications, leading to improved CVD outcomes in individuals with both T2DM and Type-1 T1DM. It has been observed that the quality of care required to improve diabetes-related healthcare outcomes is complicated by the presence of comorbidities, which leads to financial loss for all stakeholders.^17^

We did measure the prevalence of five extremely common and well-documented medical and mental health disorders (depression, gastritis, asthma, anaemia, hypothyroidism), however as a result of our small sample size, we were unable to detect any statistically significant correlations, so it was not mentioned. However, it has also been discovered that people with T2DM are more likely to have depression, thyroid gland disorders, and chronic obstructive pulmonary disease (COPD).^17^ Diabetic patients with these chronic conditions also suffer from some obstacles to their self-care like physical and financial limitations, the practicalities of receiving care, and the need for social and emotional support. The presence of these chronic conditions in diabetic patients also affects their capacity to prioritize and control the condition.

Diabetic patients exhibit a notably heightened susceptibility to the development of early atherosclerotic cardiovascular disease. Clinical investigations on intensive glycemic management have largely shown no substantial improvement in cardiovascular outcomes. Diabetes frequently causes dyslipidemia, and there is compelling evidence that decreasing cholesterol improves cardiovascular outcomes even in patients with lipid profiles that don’t seem particularly noteworthy. Early research on cardiovascular mortality in Type-1 diabetes revealed that risk doesn’t start to rise dramatically until nephropathy develops, which also happens to be accompanied by a major decline in blood pressure and lipid profile.^5^ We discovered a very substantial correlation between diabetes mellitus, dyslipidemia, and hypertension.

Several clinical guidelines advise high-risk individuals to maintain strict control of their dyslipidemia.^18^ Other studies have reported that achieving better lipid levels is easier to achieve than blood pressure or glycemic targets, especially in diabetic patients with established CHD.^19^,^20^ Triglycerides and CHD are strongly correlated in both Type-1 and Type-2 diabetes. Another study has reported that high serum triglycerides herald the development of T2DM, mainly when related to other features of metabolic syndrome or CHD but once diabetes has developed they continue to predict CHD risk, often independently of other risk factors.^21^ According to the findings of the Action to Control Cardiovascular Risk in Diabetes (ACCORD) study triglyceride, persistently low HDL, and mean LDL cholesterol below 2.1 mmol/L in diabetics eventually lead to more cardiovascular events.^22^

The HbA1c levels are a significant marker of the average three months of glucose and it has been always used to monitor, manage, or even diagnose both Type-1 and 2 diabetes.^21^ In a series of 12,785 male diabetic patients, Khan, Ola, Alhomida, Sobki, Khan 23 have shown that the HbA1c cut-point of 6.5% was associated with 3.78% false-negative predictions^12^ while the majority of the false-negative patients had borderline fasting plasma glucose (FPG) (7.0-8.0 mmol/L) and HbA1c (6.0%–6.5%), and therefore belonged to an at-risk category based on HbA1c alone criteria. These findings suggest that the status of individuals with HbA1c between 6.0% and 6.5% should be verified by combined FPG and HbA1c criteria.^23^

Similar to our study’s findings, there was no connection between a person’s HbA1c level and the onset of diabetes. It was also reported that the risk of cardiovascular mortality in T1DM patients does not considerably rise until nephropathy develops, which is associated with a marked decline in lipid profile and blood pressure.^5^ In patients with T2DM, achieving a glycemic target of an HbA1c level of 7% lowers the likelihood of developing microvascular problems and CVD. In order to avoid serious hypoglycemia during pregnancy, an HbA1c target of 6% may be ideal. Similarly in the geriatric age group people, with a history of severe microvascular or macrovascular problems, multiple co-morbidities, or long-standing T2DM may benefit from a higher level of HbA1c targeting 8 %.^24^ Diabetes frequently leads to dyslipidemia, a condition characterised by abnormal lipid levels. Extensive data supports the notion that reducing cholesterol levels significantly enhances cardiovascular outcomes, even among persons exhibiting lipid profiles that are within the expected range.

### Strengths and limitations of the study:

As far as we are aware, this is the first study of its kind conducted among patients with T2DM and comorbidities in a primary care setting in Riyadh, Saudi Arabia. The data exhibits a high level of quality, as it is derived from the Electronic System for Integrated Health Information (e-SiHi), which is an electronic application of patient data in King Saud Medical City Hospital This study has limited generalizability because of the small sample size, which was not a wide-ranging depiction of the population of patients with T2DM with comorbidities in Saudi Arabia. There is also a chance of selection bias, as the data was collected only at family medicine clinics of one tertiary care hospital in Riyadh. Also, some information on the diagnosis of co-morbidities, laboratory investigations, and body mass index measurements may be missing from patient charts.

## CONCLUSION

Regardless of gender, DM is strongly linked to hypertension and weight gain. Cholesterol and HbA1c have a positive correlation in people with DM. Patients with T2DM who are obese have been advised to lose weight. We discovered that the DM patients had a considerably larger proportion of individuals who exercised regularly, regardless of weight fluctuations. Blood pressure needs to be kept under control in DM patients. To achieve all these goals primary care physicians (PCP) are the cornerstone to facilitate this multidisciplinary care. This multidisciplinary team approach should focus on integrated management with multiple treatment goals including glucose, lipids, blood pressure control, lifestyle management, and screening for diabetes mellitus morbidities.

### Authors’ Contribution:

**KS & SIK:** Conceived, designed, collected the data, prepared initial manuscript and are responsible for integrity of research.

**AA:** Carried out statistical analysis and editing of manuscript.

**FAA:** Conducted review and final approval of manuscript.

## References

[1] Baig M, Gazzaz ZJ, Bakarman MA, Alzahrani SH (2021). Correlation of Serum Vaspin, Omentin-1, and adiponectin with metabolic phenotypes in Type-2 diabetes mellitus patients. Pak J Med Sci.

[2] Mozaffarian D, Benjamin EJ, Go AS, Arnett DK, Blaha MJ, Cushman M (2016). Heart Disease and Stroke Statistics-2016 Update:A Report From the American Heart Association. Circulation.

[3] Pfeffer MA, Claggett B, Diaz R, Dickstein K, Gerstein HC, Køber LV (2015). Lixisenatide in Patients with Type 2 Diabetes and Acute Coronary Syndrome. N Engl J Med.

[4] Roglic G (2016). WHO Global report on diabetes:A summary. Int J Noncommunicable Dis.

[5] Alotaibi A, Perry L, Gholizadeh L, Al-Ganmi A (2017). Incidence and prevalence rates of diabetes mellitus in Saudi Arabia:An overview. J Epidemiol Global Health.

[6] Karim A, Ogbeide DO, Siddiqui S, Al-Khalifa IM (2000). Prevalence of diabetes mellitus in a Saudi community. Saudi Med J.

[7] DeFronzo RA, Ferrannini E, Groop L (2015). Type 2 diabetes mellitus. Nature Rev Dis Primers.

[8] Gaede P, Vedel P, Larsen N, Jensen GVH, Parving H-H, Pedersen O (2003). Multifactorial Intervention and Cardiovascular Disease in Patients with Type 2 Diabetes. N Engl J Med.

[9] Qari FA (2005). Glycemic Control among diabetics at a university and Erfan private hospital. Pak J Med Sci.

[10] Supper I, Bourgueil Y, Ecochard R, Letrilliart L (2017). Impact of multimorbidity on healthcare professional task shifting potential in patients with type 2 diabetes in primary care:a French cross-sectional study. BMJ Open.

[11] Willett WC, Manson JE, Stampfer MJ, Colditz GA, Rosner B, Speizer EF (1995). Weight, weight change, and coronary heart disease in women:risk within the'normal'weight range. JAMA.

[12] Sherwani SI, Khan HA, Ekhzaimy A, Masood A, Sakharkar MK (2016). Significance of HbA1c test in diagnosis and prognosis of diabetic patients. Biomarker Insights.

[13] Gliklich R, Leavy M, Dreyer N (2019). Tools and Technologies for Registry Interoperability, Registries for Evaluating Patient Outcomes:A User's Guide, Addendum 2.(Prepared by L&M Policy Research, LLC under Contract. No. 290-2014-00004-C.) AHRQ Publication No. 19 (20)-EHC017-EF.

[14] Mingrone G, Panunzi S, De Gaetano A, Guidone C, Iaconelli A, Capristo E (2021). Metabolic surgery versus conventional medical therapy in patients with type 2 diabetes:10-year follow-up of an open-label, single-centre, randomised controlled trial. Lancet.

[15] Steven S, Hollingsworth KG, Small PK, Woodcock SA, Pucci A, Aribisala B (2016). Weight loss decreases excess pancreatic triacylglycerol specifically in type 2 diabetes. Diabetes Care.

[16] Cosentino F, Grant PJ, Aboyans V, Bailey CJ, Ceriello A, Delgado V (2020). 2019 ESC Guidelines on diabetes, pre-diabetes, and cardiovascular diseases developed in collaboration with the EASD:The Task Force for diabetes, pre-diabetes, and cardiovascular diseases of the European Society of Cardiology (ESC) and the European Association for the Study of Diabetes (EASD). Europ Heart J.

[17] Nowakowska M, Zghebi SS, Ashcroft DM, Buchan I, Chew-Graham C, Holt T (2019). The comorbidity burden of type 2 diabetes mellitus:patterns, clusters and predictions from a large English primary care cohort. BMC Med.

[18] Board JBS (2014). Joint British Societies'consensus recommendations for the prevention of cardiovascular disease (JBS3). Heart.

[19] Gyberg V, De Bacquer D, De Backer G, Jennings C, Kotseva K, Mellbin L (2015). Patients with coronary artery disease and diabetes need improved management:a report from the EUROASPIRE IV survey:a registry from the EuroObservational Research Programme of the European Society of Cardiology. Cardiovascular diabetology.

[20] Yudkin J, Richter B, Gale E (2010). Intensified glucose lowering in type 2 diabetes:time for a reappraisal. Springer.

[21] Sone H, Tanaka S, Tanaka S, Iimuro S, Oida K, Yamasaki Y (2011). Serum level of triglycerides is a potent risk factor comparable to LDL cholesterol for coronary heart disease in Japanese patients with type 2 diabetes:subanalysis of the Japan Diabetes Complications Study (JDCS). J Clin Endocrinol Metabol.

[22] Group AS (2010). Effects of combination lipid therapy in type 2 diabetes mellitus. N Engl J Med.

[23] Khan HA, Ola MS, Alhomida AS, Sobki SH, Khan SA (2014). Evaluation of HbA1c criteria for diagnosis of diabetes mellitus:a retrospective study of 12 785 type 2 Saudi male patients. Endocrine Res.

[24] Ismail-Beigi F, Moghissi E, Tiktin M, Hirsch IB, Inzucchi SE, Genuth S (2011). Individualizing glycemic targets in type 2 diabetes mellitus:implications of recent clinical trials. Ann Intern Med.

